# Effects of Waxy Maize Starch and Malate-Debranched Waxy Maize Starch on Gut Microbiota of Humans In Vitro and Mice In Vivo

**DOI:** 10.3390/microorganisms13092218

**Published:** 2025-09-22

**Authors:** Zhonglin Zhao, Wei Liu, Lulu Wu, Guoyu Yang, Yizhe Yan, Xiaolong Ji

**Affiliations:** 1 College of Sciences, Henan Agricultural University, Zhengzhou 450002, China; nankai20102015@126.com (L.W.); yangguoyulxy@henau.edu.cn (G.Y.); 2 Zhejiang Provincial Key Laboratory of Agricultural Microbiomics, Institute of Plant Protection and Microbiology, Zhejiang Academy of Agricultural Sciences, Hangzhou 310021, China; biolwei@sina.com; 3 College of Food and Bioengineering, Zhengzhou University of Light Industry, Zhengzhou 450000, China; anyizhe@zzuli.edu.cn; 4 National & Local Joint Engineering Research Center of Cereal-Based Foods (Henan), Zhengzhou 450001, China

**Keywords:** in vitro fermentation, waxy maize starch, malate-debranched waxy maize starch, gut microbiota, SCFAs

## Abstract

The gut microbiota plays a pivotal role in host health. Dietary components such as waxy maize starch (WMS) and malate-debranched WMS (MADBS) may serve as modulators of microbial composition and function. In this study, the effects of WMS and MADBS on murine gut microbiota in vivo and human fecal in vitro fermentation were investigated. The results of gut microbiota in mice revealed that WMS increased the abundance of *Muribaculaceae* and *Bifidobacterium*, while MADBS enriched *Ileibacterium*, *Muribaculaceae*, and *Dubosiella*. The in vitro fermentation model demonstrated that WMS increased the abundance of *Bifidobacterium*, *Lactobacillus*, *Megamonas*, and *Megasphaera*, whereas MADBS enhanced *Weissella*, *Lactobacillus*, and *Prevotella*. Both compounds decreased the levels of *Escherichia-Shigella*. Metabolically, compared to the control group, WMS improved the production of acetic, propionic, butyric, and valproic acids, while MADBS decreased the concentrations of all short-chain fatty acids (SCFAs). Compared to the control group, WMS reduced the production of CH_4_, NH_3_, and H_2_S while increasing CO_2_ yield. MADBS reduced the generation of CH_4_, NH_3_, H_2_S, and CO_2_. These findings suggest that WMS and MADBS can modulate the gut ecosystem by selectively promoting probiotics, inhibiting pathogens, and altering metabolic profiles.

## 1. Introduction

Starch is a renewable and biodegradable plant resource. The processing of food, cosmetics, and feed extensively utilizes starch and its derived compounds. Corn starch accounts for a high proportion of the global starch market. Waxy maize starch (WMS) exhibits significantly different physical and chemical properties compared with ordinary corn starch, including paste stability, transparency, and retrogradation tendency [1]. However, there are certain drawbacks to using natural waxy corn starch, such as heat resistance, acidity, and shear resistance, including its susceptibility to aging and retrogradation. It is unable to satisfy the processing and storage requirements of certain products due to these limitations [2]. Furthermore, starch is the most important carbohydrate and the main energy source in human life. However, long-term consumption of starchy products is associated with various chronic diseases [3]. Therefore, researchers have attempted to modify natural starch through different methods to improve its properties [4,5].

Debranching is a common technique for starch modification. Numerous novel qualities are possessed by debranched starch, including excellent hydrogel characteristics [6], barrier properties [7], and good mobility [8]. The unique properties allow for its use in coating and the controlled release of pharmaceuticals. In earlier research, we prepared malate-debranched WMS (MADBS) through debranching and malate esterification, which demonstrated a greater degree of substitution (DS), a higher content of resistant starch (RS) with more amorphous regions, and molecular aggregation [9]. MADBS exhibits a significantly increased RS content compared to native and singly modified starches due to its enhanced resistance to enzymatic hydrolysis. MADBS also shows superior resistance to thermal processing, acid conditions, and mechanical shear, making it more stable and suitable for industrial food applications. Due to the waxy maize origin, MADBS retains a soft texture and favorable mouthfeel, unlike high-amylose RS, which often yields firmer, less palatable textures. These unique features support the novelty and functional superiority of MADBS over conventional resistant starches.

MADBS represents a newly developed form of resistant starch, pioneered by our research team, with no existing studies evaluating its efficacy. The potential of its novel structure to impart superior or unique prebiotic functions remains uncertain, particularly in terms of modulating specific bacterial taxa such as *Bifidobacterium* and *Lactobacillus*, and reducing harmful metabolites like ammonia or certain short-chain fatty acids (SCFAs). To assess whether MADBS enhances the abundance of beneficial bacteria and promotes the production of advantageous SCFAs, we employed an in vitro fermentation model to compare the effects of MADBS with those of wheat maize starch (WMS) on microbial composition and metabolomic profiles. The in vitro fermentation bioassay is a well-established methodology for assessing the impact of dietary components on gut microbiota, offering a valuable alternative to in vivo studies and facilitating the exploration of interactions between gut microbiota and dietary substrates. Comprehensive analyses were conducted on changes in gut microbiota, gas production, and SCFA levels. Additionally, the effects of WMS and MADBS on the intestinal microbiota of mice were investigated. The primary aim of this study was to provide insights into the application and potential benefits of MADBS.

## 2. Experimental Section

### 2.1. Fermentation Medium

WMS and malate MADBS were prepared according to the previously published method [9]. The anaerobic yeast extract–casein hydrolysate–fatty acid (YCFA) medium served as the fermentation medium for control [10]. The YCFA medium contains tryptone (1%), yeast extract (0.25%), L-cysteine (0.1%), heme solution (0.01%), NaCl (0.09%), KH_2_PO_4_ (0.045%), K_2_HPO_4_ (0.045%), MgSO_4_·7H_2_O (0.09%), CaCl_2_·6H_2_O (0.006%), vitamin I solution (200 µL), resazurin solution (1.0 mL), and pH 6.5. Instead, 8 g/L of WMS or MADBS was used in the respective cultures. Due to their limited solubility, WMS and MADBS were added to the medium prior to autoclaving. The autoclaved mixtures were then incubated in an anaerobic workstation for 24 h. The medium was autoclaved for 20 min at 121 °C.

### 2.2. Collection of Human Fecal Samples

This study included nine healthy adults aged between 23 and 43. All donors had a normal diet and had not taken any medications for the last four months before sample collection. Nine healthy adult donors provided fecal samples following a standardized protocol. Donors were instructed to collect fresh samples in sterile containers, which were immediately transported to the laboratory and processed to make the bacteria suspension within 1 h to minimize microbial community changes. Each sample (1.0 g) was resuspended separately in 10 mL PBS buffer (phosphate-buffered saline, pH 7.0), and the fecal slurry was filtered through 200 mesh filter to remove debris. Samples from each donor were pooled and were injected into the fermentation bottle. The experiment complied with all the ethical requirements. Ethical approval for this study was granted by the Ethics Committee of the Zhejiang Academy of Agricultural Sciences (approval code: ZAASES [2024]01, and approval date: 25 November 2024).

### 2.3. In Vitro Fermentation

The fecal suspensions were inoculated into YCFA, WMS, and MADBS media. In vitro fermentation was performed at 37 °C for 24 h. In addition, 8 mg/mL of WMS and MADBS was used for the in vitro fermentation procedure. The concentration was selected in in vitro experiments based on our previous dose-gradient studies with other prebiotics. This concentration represented the maximum tolerable dose. In the anaerobic chamber, 500 μL of freshly prepared fecal suspension inoculum was introduced into a 15 mL anaerobic bottle containing 5.0 mL of YCFA medium or control medium, respectively, using sterile disposable syringes. Fermentation was carried out at 37 °C for 24 h. All experiments were performed in triplicate to ensure reproducibility. Throughout the fermentation process, oxygen was excluded, and the pH of the YCFA medium was maintained at 6.5. All fermentations were performed under static anaerobic conditions, with the anaerobic headspace maintained by pure nitrogen gas.

### 2.4. Determination of Gas Composition

After a 24 h fermentation period, the butyl rubber stopper was punctured inside a biological safety cabinet using a sterile syringe needle. As described in the literature, the gas generated during fermentation inside the vial was then introduced into the gas analyzer (Empaer gas detector, Shenzhen, China), which could measure the content of five gases simultaneously through the principle and working of a sensor. The infrared detector was used to determine methane (CH_4_) and carbon dioxide (CO_2_), while ammonia (NH_3_), hydrogen (H_2_) and hydrogen sulfide (H_2_S) were monitored using electrochemical sensors. A needle-tipped gas-impermeable syringe was inserted into the rubber cap of each vessel to measure the concentration of each gas produced after being incubated at 37 °C for 24 h. The results were expressed in liters of gas.

### 2.5. Short-Chain Fatty Acid (SCFA) Quantification

A solution of crotonic acid (0.6464 g) in 2.5% (*w*/*v*) metaphosphoric acid (100 mL) was prepared. Then, 0.5 mL of fermentation broth was added to 0.1 mL of crotonic acid metaphosphoric acid, and the solution was acidified for more than 24 h at −20 °C before being subjected to gas chromatography (GC) analysis. The fermentation broth was centrifuged at 13,000 rpm for 3 min, and the supernatant was filtered for the detection of the SCFA concentration. The concentrations of SCFAs were determined using GC (Shimadzu, GC-2010 Plus, Kyoto, Japan). GC analysis was carried out using a 0.32 mm × 30 m × 0.5 µm DB-FFAP column (Agilent Technologies, Santa Clara, CA, USA). Trans-2-butenoic acid was used as an internal standard.

The GC operating conditions were as follows: high-purity nitrogen carrier gas was maintained at a flow rate of 12 mL/min. The detector gas mixture comprised nitrogen (30 mL/min), hydrogen (40 mL/min), and air (400 mL/min). The temperature program initiated with a 70 °C isothermal hold for 30 s, followed by ramping to 190 °C, and subsequently increasing to 240 °C at 40 °C/min (5 min hold). The FID detector and vaporization chamber were maintained at 250 °C and 280 °C, respectively.

### 2.6. Analysis of Intestinal Flora

Genomic DNA extracted from fecal samples (FastPure Stool DNA Isolation Kit, MJYH, Shanghai, China) was assessed for purity using a NanoDrop 2000 spectrophotometer (Thermo Scientific, Wilmington, DE, USA). The 16S rRNA gene was amplified with primer pairs 338F (5′-ACTCCTACGGGAGGCAGCAG-3′) and 806R (5′-GGACTACHVGGG- TWTCTAAT-3′). Purified amplicons were pooled in equimolar amounts, and paired-end sequencing was performed on an Illumina NextSeq2000 platform (Illumina, San Diego, CA, USA) according to the standard protocols by Majorbio Bio-Pharm Technology Co., Ltd. (Shanghai, China). The raw sequencing reads were deposited into the sequence read archive database of National Center for Biotechnology Information. Processing of raw sequencing data involved de-multiplexing (in-house Perl script), quality filtering (fastp v0.19.6 [11]), and merging (FLASH v1.2.7 [12]). Then, the optimized sequences were clustered into operational taxonomic units (OTUs) using UPARSE (version 7.1) [13,14]. Representative sequences for each OTU were defined by selecting the most abundant sequence.

Selection of the representative read for each OTU was performed utilizing the QIIME ackage (version 1.9). With a confidence threshold of 0.7, OTUs were taxonomically annotated using the RDP Classifier and the SILVA database (v138) as a reference. The community structure of bacteria was analyzed at the genus and phylum levels based on the taxonomic information. Alpha diversity was calculated based on observed OTU, and the Chao index. Principal component analysis (PCA) and principal co-ordinates analysis (PCoA) were performed to compare the similarities and differences in microbial communities among the CK, WCS, and MADBS groups. Alpha diversity comparisons among the three groups were performed using between-group difference testing of the Chao1 index, analyzed by one-way ANOVA. R (version 3.3.1) was used for statistical analysis and graphing.

### 2.7. Animal Experiment Design

A total of 30 C57BL/6 healthy male mice (8 weeks old) were purchased from Hangzhou Ziyuan Laboratory Animal Technology Co., Ltd., Hangzhou, China, and then randomly divided into three groups: a control group (CK), a WMS group receiving 40 mg per day, and a MADBS group receiving 40 mg per day. Each group contained 10 mice. The animals were randomly allocated to cages across a room to account for spatial variability. Each mouse was placed separately in a sterile animal chamber, maintaining the room temperature at approximately 20 °C and the relative humidity between 45% and 55%. The mice were fed individually in their own cages. All the groups were administered for 14 days and underwent the same experimental procedure with a regular diet. The fresh fecal samples were collected immediately after defecation to analyze intestinal flora. The mice were euthanized by cervical dislocation after being anesthetized with isoflurane. For the gut microbiota analysis, 5 mice in the CK group and 8 mice in the treatment group were included in the analysis. Due to the difficulty in dissolving WCS and MADBS, during the initial gavage procedure, 9 mice experienced pulmonary aspiration and died. Consequently, 5 mice from the CK group were reassigned to the treatment groups to compensate for the loss. The Zhejiang Academy of Agricultural Sciences’ Ethics Committee approved this research (No. 2024ZAASLA111).

### 2.8. Correlation Analysis of Metabolite Factors and Gut Microbiota

Correlation heatmaps were generated based on correlation coefficients between the gut microbiota and both gases and SCFAs. The numerical matrix was presented through a heatmap. The color gradient in the heatmap reflects the data information in the two-dimensional matrix or table, with color intensity representing the magnitude of data values, thereby providing an intuitive visualization where numerical values are represented by defined color shades. The analysis was conducted using R software (version 3.3.1) with the pheatmap package (version 1.0.12).

### 2.9. Statistical Analysis

Statistical analysis was performed using GraphPad Prism software (version 9.5). Statistical differences were determined by conducting either a paired *t*-test or ANOVA. Data are presented as mean ± SD. The variable importance in projection (VIP) in OPLS-DA (VIP > 1) and the *p*-value (*p* < 0.05) were further used to identify significant differential metabolites.

## 3. Results

### 3.1. Effects of WMS and MADBS on SCFA Production

**Figure 1:**
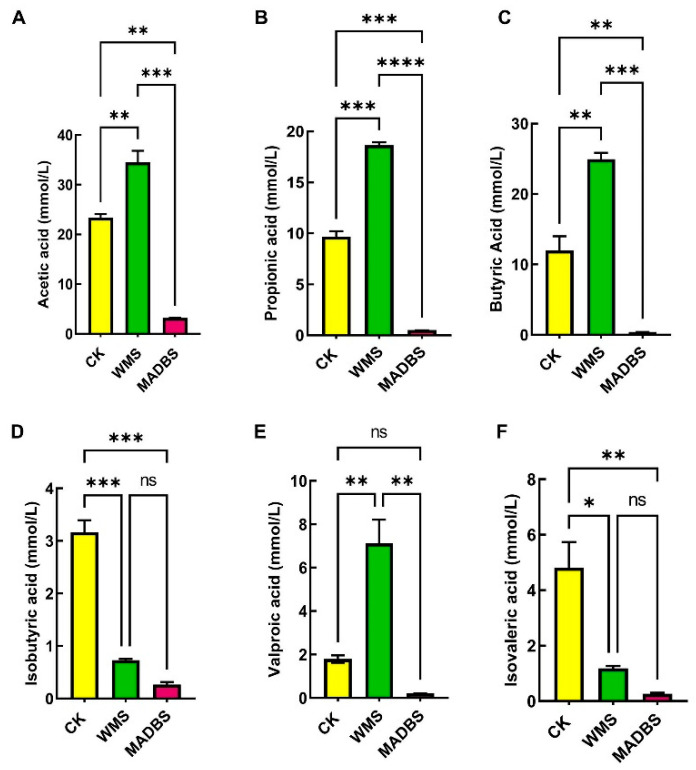
In vitro fermentation results in the SCFA content in the fermentation broth. (**A**) Acetic acid, (**B**) propionic acid, (**C**) butyric acid, (**D**) isobutyric acid, (**E**) valeric acid, and (**F**) isovaleric acid. * *p* < 0.05, ** *p* < 0.01, *** *p* < 0.001, and **** *p* < 0.0001. ns: not significant.

WMS and MADBS in human fecal samples were fermented to produce SCFAs (Figure 1, Supplementary Materials Table S1). Acetic, propionic, butyric, and valproic acid concentrations were notably higher in the WMS group than in the CK group (Figure 1). Among the three groups, the MADBS group had the lowest SCFA levels.

### 3.2. Analysis of the Content of Gas Component

**Figure 2:**
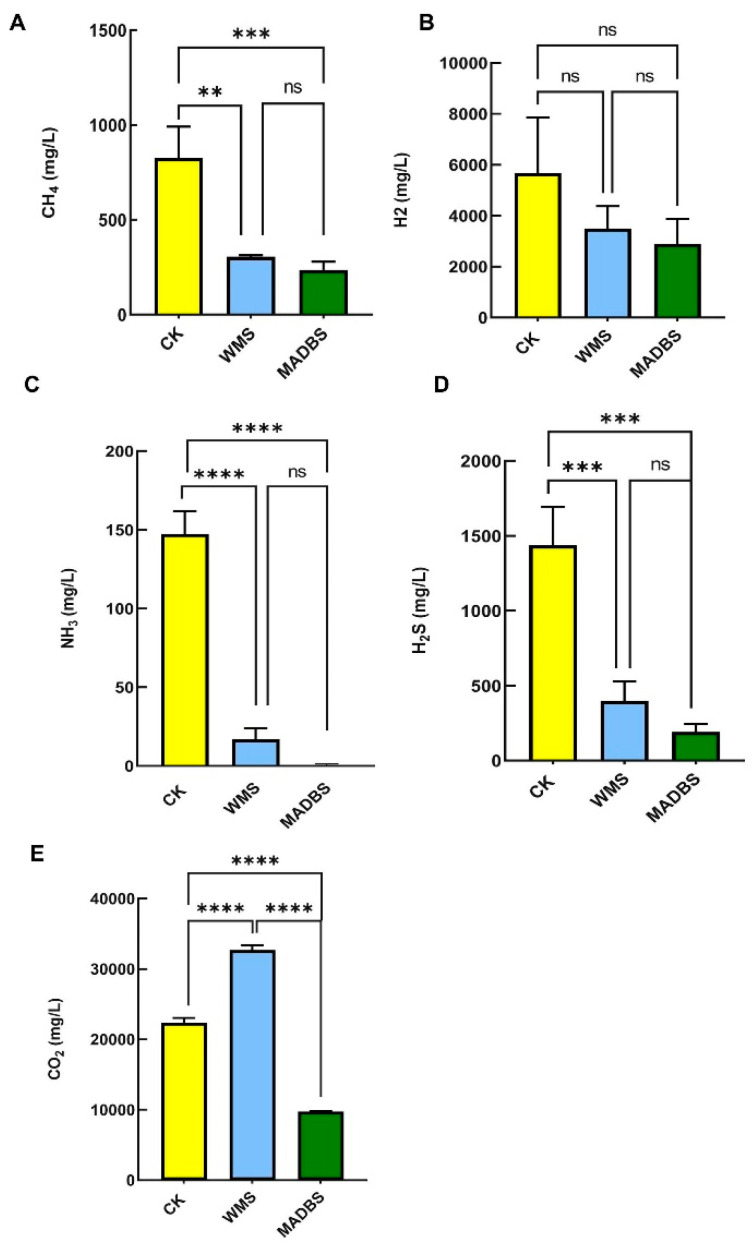
Gas changes after in vitro human fecal fermentation. (**A**) CH_4_, (**B**) H_2_, (**C**) NH_3_, (**D**) H_2_S, and (**E**) CO_2_. ** *p* < 0.01, *** *p* < 0.001 and **** *p* < 0.0001. ns: not significant.

After 24 h of in vitro human fecal fermentation, gas component analysis revealed that the CK group exhibited higher levels of CH_4_, NH_3_, H_2_, and H_2_S than the WMS and MADBS groups. However, the WMS group had the highest level of CO_2_ level of the three groups. Additionally, the MADBS group significantly reduced the generation of CH_4_, NH_3_, H_2_S, and CO_2_ compared to the WMS and YCFA groups (Figure 2, Supplementary Materials Table S2). This suggests that MADBS and WMS have the potential to regulate gas production.

### 3.3. Impact of WMS and MADBS on Human Gut Microbiota by In Vitro Fermentation

**Figure 3:**
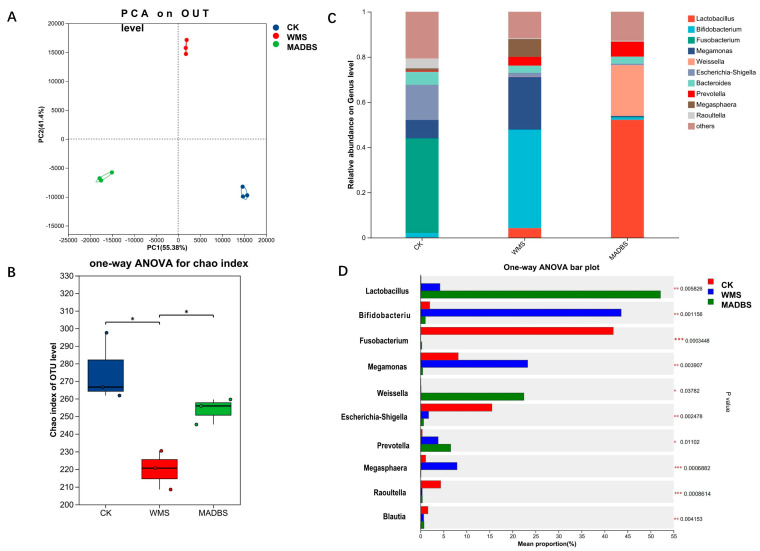
The results of human gut microbiota after in vitro fecal fermentation. (**A**) PCA on OTU level of the gut microbial community differences between CK, WMS, and MADBS groups. (**B**) The difference in the Alpha Diversity index among CK, WMS, and MADBS groups. * *p* < 0.05. (**C**) Community barplot analysis on genus level. (**D**) Genera with significant abundance differences among CK, WMS, and MADBS samples. A one-way ANOVA was used to evaluate the significance of differences between the indicated groups. * *p* < 0.05; ** *p* < 0.01; *** *p* < 0.001.

To investigate the impact of WMS and MADBS on human gut microbiota structure, we analyzed the microbial composition of fecal samples using in vitro fermentation. The PCA plot shows microbial community profiles of a pooled fecal sample from nine healthy donors after 24 h of fermentation under three conditions (CK, WMS, MADBS). Each condition included three technical replicates, represented by the nine points (three points per condition). The microbial composition of WMS and MADBS groups deviated from the cluster of control according to the PCA analysis (Figure 3A). WMS and MADBS treatment exerted a notable impact on the bacterial community’s beta diversity. Comparison of alpha diversity indicated that WMS and MADBS changed the gut microbial community diversity (Figure 3B).

Relative abundance analysis at the genus level revealed that WMS stimulated the growth of *Bifidobacterium, Lactobacillus*, *Megamonas*, and *Megasphaera*, whereas *Escherichia-Shigella* was found to be more abundant in the CK group (Figure 3C). These results suggest that WMS may modulate the gut microbiota and enhance the growth of probiotics. The results also demonstrated that the MADBS group had a significantly higher abundance of *Weisslla*, *Lactobacillus*, and *Prevotella* than the other two groups. However, the MADBS group had reduced levels of *Fusobacterium*, *Escherichia-Shigella*, and *Megamonas*.

Microbial communities showing significant differences among the three groups were statistically analyzed. The 10 dominant genera are shown in Figure 3D. The CK group was significantly enriched in *Fusobacterium*, *Escherichia-Shigella*, *Raoultella*, and *Blautia*. In contrast, the WMS group showed increased abundances of *Lactobacillus*, *Bifidobacterium*, *Megamonas*, and *Prevotella*, while the MADBS was dominated by *Lactobacillus*, *Weisslla*, and *Prevotella*. Table 1 summarizes the 10 statistically significant differences observed at the genus level across all three groups. A total of 54 bacterial genera showed significant differences (Supplementary Materials Table S3).

**Figure 4:**
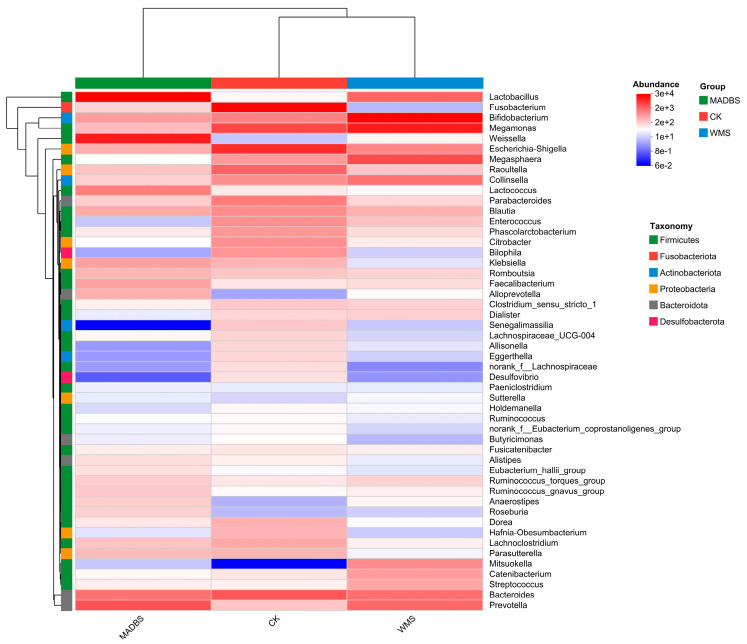
Community heatmap analysis at the genus level.

The heatmap illustrates the distribution of the top dominant species in three groups, demonstrating the trends of species changes in the CK and treatment groups. The changes in abundance of different species were displayed using the gradient of color blocks, with the values corresponding to the color gradient. The dominant species in the MADBS group were *Lactobacillus*, *Weissella*, *Lactococcus*, *Klebsiella*, *Faecalibacterium*, *Alloprevotella*, *Anaerostipes*, *Roseburia*, and *Klebsiella*, while the abundance of *Senegalimassilia*, *Bilophila*, *Desulfovibrio*, *Allisonella*, *Eggerthella*, and *Lachnospiraceae* was very low when compared to the CK group (Figure 4). The dominant species in the WMS group were *Lactobacillus*, *Bifidobacterium*, *Megamonas*, *Megasphaera*, *Collinsella*, *Mitsuokella*, *Catenibacterium*, *Streptococcus*, and *Prevotella*. However, the abundance of *Lachnospiraceae*, *Desulfovibrio*, *Fusobacterium*, *Bilophila*, *Butyricimonas*, *Roseburia,* and *Hafnia-Obesumbacterium* was lower than that in the CK group.

### 3.4. Results of the Correlation Analysis of Metabolite Factors and Gut Microbiota

**Figure 5:**
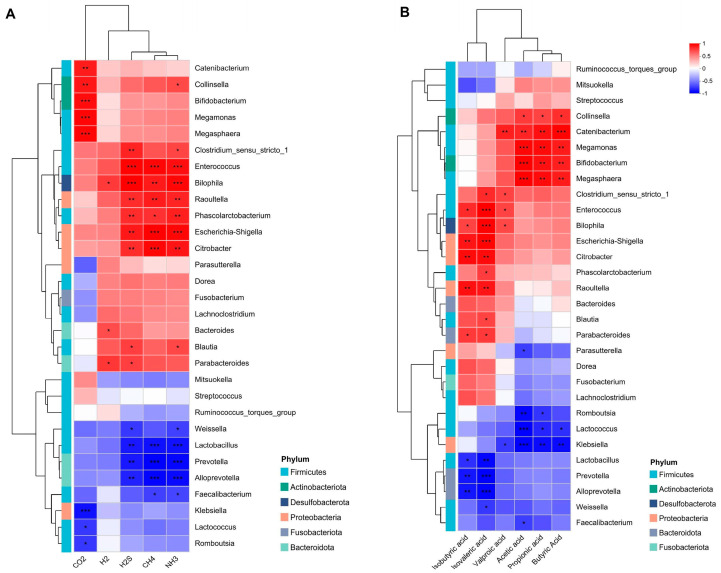
Environmental factor heatmap. (**A**) The heatmap displays the correlation between the gut microbiota and gases. (**B**) The correlation between metabolite SCFA and the gut microbiota. Color depth represents the R-value magnitude (see legend for value intervals). Statistical significance is indicated as follows: * *p* < 0.05; ** *p* < 0.01; *** *p* < 0.001.

We investigated the association between the gut microbiome population and the production of gas and SCFA compounds. NH_3_, CH_4_, and H_2_S exhibited positive correlations with the high levels of *Enterococcus*, *Bilophila*, *Raoultella*, *Phascolarctobacterium*, *Escherichia-Shigella*, and *Citrobacter*. While it demonstrated a negative correlation with *Lactobacillus*, *Prevotella*, and *Alloprevotella* (Figure 5A), CO_2_ was positively correlated with the high levels of certain species, such as *Megamonas*, *Megasphaera*, *Bifidobacterium*, *Collinsella*, and *Catenlbacterium*.

The isobutyric and isovaleric acids exhibited positive correlations with *Enterococcus*, *Bilophila*, *Escherichia-Shigella*, *Citrobacter*, *Raoultella*, and *Parabacteroides* and a negative correlation with the abundance of *Lactobacillus*, *Prevotella*, and *Alloprevotella* (Figure 5B). The contents of acetic, propionic, and butyric acids were positively correlated with the abundance of *Collinsella*, *Catenibacterium*, *Megamonas Megasphaera*, and *Bifidobacterium.* The relative abundance of *Romboutsia*, *Lactococcus*, and *Klebsiella* demonstrated a negative correlation with the acetic acid content. Valproic acid levels showed a positive correlation with the abundance of *Catenibacterium*, *Enterococcus*, *Bilophila*, and *Clostridium_sensu_stricto_1*.

### 3.5. Analysis of Gut Microbiota in Mice

**Figure 6:**
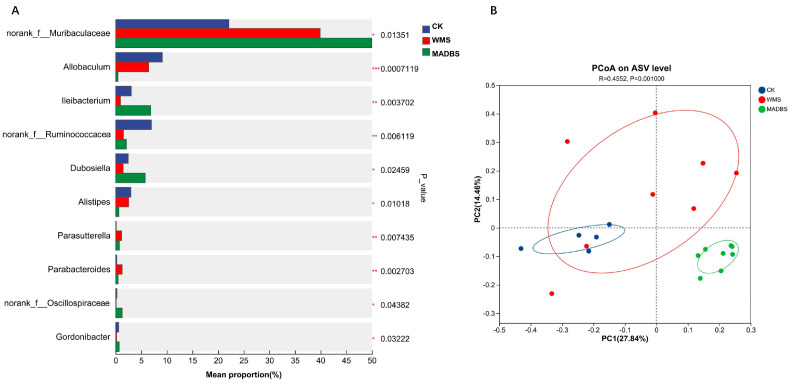
Results of gut microbiota in mice. (**A**) The top 10 abundant genera in the CK, WMS, and MADBS groups. The vertical axis represents the genus names at various classification levels, and the horizontal axis represents the abundance percentage of the samples. * *p* < 0.05, ** *p* < 0.01, *** *p* < 0.001. (**B**) PCoA plot of microbial communities (mice, n = 5 for CK; n = 8 for WMS and MADMS).

The gut microbiota analysis of mice revealed that the WMS and MADBS groups differed from the CK group. Figure 6A illustrates the top 10 abundant genera with variations in abundance between the CK, WMS, and MADBS groups. Relative abundance analysis of the mouse gut microbiota at the genus level indicated that the WMS treatment promoted the abundance of norank_f_*Muribaculaceae*, *Parasutterella* and *Parabacteroides* while decreasing that of *Allobaclum*, *Ileibacterium*, *Alistipes*, norank_f_*Ruminococcaceae*, norank_f_*Oscillospiraceae*, *Gordonibacter*, and *Dubosiella* when compared to the CK group. The relative abundance of *Allobaclum*, *Alistipes*, and norank_f_*Ruminococcaceae* was reduced in the MADBS group. In contrast, the abundance of *Ileibacterium,* norank_f_*Muribaculaceae*, norank_f_*Oscillospiraceae*, *Dubosiella*, and *Gordonibacter* was significantly higher in the MADBS group than in the other two groups. PCoA analysis revealed significant differences in the community structures of the gut microbiotas among the CK, WMS, and MDB groups in mice (Figure 6B). The gut microbiota of mice in MADBS was separated from other two groups. Table 2 summarizes the top 10 statistically significant differences observed at the genus level across all three groups. Thirty bacterial genera showed significant differences among the three groups (Supplementary Materials Table S4).

## 4. Discussion

Debranched starch (DBS) is primarily composed of linear starch molecules formed by the debranching of WMS, while MADBS is produced through the esterification reaction between MA (malic acid) and the hydroxyl groups at the C2, C3, and C6 positions of the glucose units in DBS. MADBS exhibited a higher degree of substitution (DS) and a greater content of resistant starch (RS) [9]. This was accompanied by an increase in amorphous regions and molecular aggregation. WMS and MADBS can be degraded into small molecular substances by intestinal microorganisms through anaerobic fermentation, including SCFA and gas. SCFAs serve as a link between the host and the gut microbiota. SCFAs play a crucial role in maintaining the normal function and morphology of the large intestine [15]. By lowering the intestinal pH, they effectively prevent the growth of pathogenic bacteria [16]. In vitro fermentation results revealed that the WMS group yielded significantly higher concentrations of acetic, propionic, butyric, and valproic acids than the CK group, whereas the MADBS group produced the lowest overall SCFA levels. The brain absorbs acetic acid, which diminishes appetite and alleviates intestinal inflammation [17]. Butyric acid can attenuate intestinal inflammation and delay the progression of chronic kidney disease [18,19]. Propionic acid exerts antibacterial effects by increasing the secretion of antimicrobial peptides within the host organism [20]. According to research, valproic acid is an anticonvulsant drug, which, along with other SCFAs, exerts inhibitory effects on cancer cell proliferation by modulating various signaling pathways [21].

The decrease in SCFA production may be attributed to ineffective microbial fermentation of MADBS. One potential explanation is that MABDS is difficult to use effectively due to its complex structure and limited microbial metabolic capacity. Our data suggest that MADBS may suppress the proliferation of SCFA-producing microbial populations, leading to decreased SCFA production. The decrease in SCFAs in the MADBS group was associated with reduced relative abundance of *Butyricimonas* and *Clostridium* (Figure 4), both of which are producers of SCFAs. The abundance of *Lactobacilli* increased, but these strains may lack a complete SCFAs synthesis pathway, leading to insufficient final product formation. The relationship between gut microbiota and SCFAs production involves intricate mechanisms that warrant further comprehensive study. Slowly fermentable substances (such as β-glucan) can form a gel-like matrix in the intestine, which lowers blood sugar and serum lipid levels [22]. Furthermore, hard-to-ferment foods can retain water, promote intestinal peristalsis, and alleviate constipation [23]. Prebiotics with rapid fermentation characteristics, such as fructooligosaccharides, tend to induce excessive flatulence and intestinal bloating [24], whereas slowly fermenting foods may exhibit a reduced propensity to cause gastrointestinal discomfort. The gas analysis results further confirmed our hypothesis, demonstrating that MADBS significantly reduced gas production (Figure 2).

Gas analysis revealed that the WMS and MADBS groups exhibited lower levels of CH_4_, NH_3_, and H_2_S. This effect was more notable in the case of MADBS. Figure 5A demonstrates that the CH_4_ and H_2_S levels showed positively correlations with the high abundances of *Bilophila*, *Raoultella*, and *Escherichia-Shigella*, all of which showed reduced abundance in the MADBS group. Conversely, these gases exhibited negative correlations with *Lactobacillus*, *Prevotella*, and *Alloprevotella,* all of which were more enriched in the MADBS group. The abundances of H_2_S-producing bacteria such as *Bilophila* and *Desulfovibrio* were decreased in the MADBS group compared to other groups (Figure 4). These shifts in microbial composition were associated with reduced gas production. Although H_2_S is an important biological mediator, its high concentrations can be potentially toxic to tissues and may contribute to colon cancer and inflammation [25,26]. Furthermore, CH_4_ may play a critical role in cardiovascular health, conditions that are often associated with the onset of diabetes and obesity [27,28].

Given the crucial role of the gut microbiota in human health and disease, its composition and abundance are subject to dietary influence. Certain foods can promote the growth of beneficial gut microbes, thereby improving the intestinal environment [15]. Microbial communities in fecal samples and their metabolic changes are frequently studied by in vitro fermentation experiments [29]. The effects of WMS and MADBS on the human gut microbiota were evaluated by an in vitro fermentation model. The results showed that WMS promoted the proliferation of intestinal *Bifidobacteria* and *Lactobacillus*, two known beneficial bacteria. *Bifidobacteria*, a key probiotic in the human intestine, are essential for protecting the gut from pathogenic infections [30]. WMS and MADBS significantly reduced the abundance of *Escherichia-Shigella*, the leading cause of human bacillary dysentery [31]. The results demonstrated that WMS significantly promotes the proliferation of probiotics while concurrently suppressing the growth of pathogenic bacteria.

The findings also demonstrated that the MADBS group exhibited a significantly higher abundance of *Weissella*, *Lactobacillus*, and *Prevotella* than the other two groups. *Weissella* species, as members of the lactic acid bacteria group, demonstrate pathogen inhibitory activity and probiotic potential [32]. Furthermore, this genus demonstrated promising therapeutic effects for atopic dermatitis and certain cancers [33,34]. *Prevotella* is one of the predominant fiber degrading bacteria in the intestine [35]. Previous studies indicated that *Prevotella* can be markedly increased by the consumption of resistant starch [36,37]. Similarly, the abundance of *Prevotella* was significantly increased by MADBS treatment. The MADBS group showed a decreased abundance of *Fusobacterium*, *Escherichia-Shigella*, and *Megamonas*. Studies indicated an association between *Megamonas* and non-alcoholic fatty liver disease in children and adolescents [38] since *Megamonas* can enhance lipid absorption and obesity [39]. Additionally, prior research has revealed that *Fusobacterium nucleatum* infection is frequently detected in human colorectal cancer and promotes colorectal carcinogenesis [40,41]. Patients with ulcerative colitis have *Fusobacterium varium* bacteria [42]. MADBS significantly increases the abundance of beneficial gut microbiota while suppressing pathogenic bacterial populations. Therefore, MADBS may represent a potential dietary strategy for gut microbiota modulation. However, translating these findings into clinical and commercial applications will require further research.

The effects of WMS and MADBS on the intestinal microbiota of mice were investigated in this study using mouse models. A species composition analysis of the mouse gut microbiota after WMS treatment revealed a significant rise in the population of *Muribaculaceae* and *Bifidobacterium*. *Muribaculaceae* is a potential probiotic bacterial family that produces SCFAs via mucin glycans and dietary fibers [43]. *Muribaculaceae* can alleviate type 2 diabetes, obesity, and inflammatory bowel disease [44,45,46]. MADBS increased the abundance of intestinal beneficial bacteria, such as *Ileibacterium* [47], *Muribaculaceae*, and *Dubosiella*. *Dubosiella newyorkensis* can modulate immune tolerance in colitis [48]. The results demonstrated that WMS and MADBS significantly enhance the abundance of beneficial gut microbiota in mice.

This study utilized an in vitro fecal fermentation model to explore the effects of WMS and MADBS on the adult gut microbiota, its metabolites, and the subsequent formation of SCFAs and gases. Notably, Liu et al. employed the Mucosal-Simulator of the Human Intestinal Microbial Ecosystem (SHIME) to evaluate the colonic fermentation profiles of WMS [49]. They demonstrated that the cooked waxy starch increased the butyrate production and enriched *Bifidobacteriaceae* abundance, which is consistent with the result obtained in our experiment. Our fermentation processes were conducted under strictly maintained anaerobic conditions. Although differences were observed in the two fecal fermentation processes, the results obtained were essentially consistent, thereby validating the feasibility of the methodology. This study synergizes in vivo rat model analyses with human fecal in vitro fermentation to elucidate diet–gut microbiota interactions, advancing understanding of dietary fiber-mediated microbial modulation.

## 5. Conclusions

WMS and MADBS significantly influence the richness and diversity of murine gut microbiota. Specifically, WMS treatment resulted in a higher relative abundance of *Muribaculaceae* and *Bifidobacterium*, whereas MADBS treatment elevated the levels of *Ileibacterium*, *Muribaculaceae*, and *Dubosiella*. In vitro fermentation experiments on human feces also demonstrated that WMS significantly promoted the growth of *Bifidobacterium*, *Lactobacillus*, *Megamonas*, and *Megasphaera*, while MADBS enhanced the proliferation of *Weissella*, *Lactobacillus*, and *Prevotella*. Notably, both treatments significantly reduced the abundance of *Escherichia-Shigella*. Metabolically, WMS improved the production of acetic, butyric, propionic, and valproic acids, whereas MADBS decreased the concentration of total SCFAs. In addition, both treatments resulted in lower levels of CH_4_, NH_3_, and H_2_S compared to the CK group.

These findings demonstrate that WMS and MADBS can effectively modulate the gut microbial ecosystem by enriching probiotics and suppressing potential pathogens while concurrently altering metabolic outputs. This suggests their potential as dietary interventions for gut microbiota regulation.

## Figures and Tables

**Table 1:** The impact of WMS and MADBS on the abundance of the top ten genera on humans’ in vitro fermentation.

Genus	CK-Mean (%)	MADBS-Mean (%)	WMS-Mean (%)	*p* Value
g_*Lactobacillus*	0.1005	52.14	4.211	0.005826
g_*Bifidobacterium*	1.984	1.033	43.6	0.001156
g_*Fusobacterium*	41.88	0.2564	0.01305	0.0003448
g_*Megamonas*	8.181	0.4594	23.28	0.003907
g_*Weissella*	0.01957	22.45	0.1051	0.03782
g_*Escherichia-Shigella*	15.51	0.6525	1.742	0.002478
g_*Prevotella*	0.366	6.548	3.821	0.01102
g_*Megasphaera*	1.096	0.08221	7.922	0.0006882
g_*Raoultella*	4.367	0.3856	0.3602	0.0008614
g_*Blautia*	1.594	0.7373	0.6747	0.004153

Mean (%) represents the average relative abundance across different groups.

**Table 2:** The impact of WMS and MADBS on the abundance of the top ten genera in mice.

Genus	CK-Mean (%)	MADBS-Mean (%)	WMS-Mean (%)	*p* Value
g__norank_f__*Muribaculaceae*	22.1	49.97	39.89	0.01351
g__*Allobaculum*	9.111	0.4608	6.461	0.0007119
g_*_Ileibacterium*	3.05	6.845	0.9708	0.003702
g__norank_f__*Ruminococcaceae*	6.984	2.117	1.526	0.006119
g__*Dubosiella*	2.477	5.766	1.471	0.02459
g__*Alistipes*	2.983	0.6399	2.547	0.01018
g__*Parasutterella*	0.143	0.7421	1.169	0.007435
g__*Parabacteroides*	0.2318	0.4862	1.285	0.002703
g__norank_f__*Oscillospiraceae*	0.2601	1.289	0.1118	0.04382
g__*Gordonibacter*	0.6055	0.7002	0.2115	0.03222

Mean (%) represents the average relative abundance across different groups.

## Data Availability

The original contributions presented in this study are included in the article/Supplementary Material. Further inquiries can be directed to the corresponding authors.

## Supplementary Materials

The following supporting information can be downloaded at: https://www.mdpi.com/article/10.3390/microorganisms13092218/s1, Table S1. SCFAs content after in vitro fermentation. Table S2. Gas content after in vitro fermentation. Table S3. The impact of WMS and MADBS on the abundance of 54 significantly differential bacterial genera. Table S4. The impact of WMS and MADBS on the abundance of 30 significantly differential bacterial genera in mice.
