# Supplementary material for: Effects of Waxy Maize Starch and Malate-Debranched Waxy Maize Starch on Gut Microbiota of Humans In Vitro and Mice In Vivo
Source: Microorganisms. 2025 Sep 22;13(9):2218. doi: 10.3390/microorganisms13092218 (PMC12472379; doi:10.3390/microorganisms13092218)
Supplement: Supplementary file 1 [file microorganisms-13-02218-s001.zip › microorganisms-3848881-supplementary.pdf]

Table S1. SCFAs content after *in vitro* fermentation.

	**Acetic acid**	**Propionic acid**	**Isobutyric acid**	**Butyric Acid**	**Isovaleric acid**	**Valproic acid**
CK	23.3753±0.6986	9.6835±0.5416	3.1661±0.2291	12.0011±2.0029	4.8152±0.9212	1.7937±0.1710
WMS	34.4686±2.3436 **	18.6523±0.2925* **	0.7330±0.0216***	24.9731±0.8854**	1.1837±0.0834*	7.1205±1.0933**
MADBS	3.2246±0.0102 **###	0.5363±0.0297** *####	0.2644±0.0493***	0.4352±0.0517**# ##	0.2602±0.0459**	0.2133±0.0249##

Data are presented as mean ± standard deviation (SD). Unit: mmol/L. * represents compared with the control group, # represents compared with the WMS group. ** P< 0.01, *** P< 0.001, ****P< 0.0001, ##P < 0.01, ### P< 0.001, and ####P < 0.0001.

Table S2. Gas content after *in vitro* fermentation.

	**CH_4**	**H_2**	**NH_3**	**H_2S**	**CO_2**
**CK**	826±165	5679±2217	147±15	1437±257	22387±151
**WMS**	306±8 **	3500±881	17±7 ****	399±129***	32700±693****
**MADBS**	236±43***	2900±977	1±1 ****	192±52***	9759±67****####

Data are presented as mean ± standard deviation (SD). Unit: mg/L. * represents compared with the control group, # represents compared with the WMS group. **P< 0.01, *** P< 0.001, ****P < 0.0001, and #### P< 0.0001.

Table S3. The impact of WMS and MADBS on the abundance of 54 significantly differential bacterial genera

Species name	CK-Mean(%)	MADBS-Mean(%)	WMS-Mean(%)	P value
g__Christensenellaceae_R-7_group	0.03197	0.001957	0.001957	0.0001479
g__Fusobacterium	41.88	0.2564	0.01305	0.0003448
g__Butyricimonas	0.08939	0.0522	0.0124	0.0004924
g__Megasphaera	1.096	0.08221	7.922	0.0006882
g__Bilophila	1.257	0.008482	0.02414	0.0007773
g__Raoultella	4.367	0.3856	0.3602	0.0008614
g__norank_f__Ruminococcaceae	0.04633	0.0124	0.0006525	0.0009176
g__Eubacterium	0.07438	0.003915	0.00261	0.001099
g__Citrobacter	1.438	0.07569	0.1416	0.001121
g__Bifidobacterium	1.984	1.033	43.6	0.001156
g__unclassified_f__Lachnospiraceae	1.233	1.383	0.2121	0.001666
g__Lachnoclostridium	0.7608	0.3902	0.1246	0.001904
g__Hafnia-Obesumbacterium	0.5729	0.04111	0.02088	0.002363
g__Escherichia-Shigella	15.51	0.6525	1.742	0.002478
g__Anaerostipes	0.01109	0.3008	0.1011	0.00333
g__Klebsiella	0.5625	0.9592	0.03915	0.003455
g__Megamonas	8.181	0.4594	23.28	0.003907
g__Blautia	1.594	0.7373	0.6747	0.004153
g__Alloprevotella	0.00783	0.6486	0.09657	0.004433
g__unclassified_k__norank_d__Bacteria	0.1703	0.009135	0.02479	0.005419
g__UCG-002	0.0398	0.02806	0.02088	0.005622
g__Lactobacillus	0.1005	52.14	4.211	0.005826
g__Eubacterium_hallii_group	0.07047	0.1873	0.04045	0.007243
g__Sellimonas	0.03262	0.01109	0.001957	0.007583
g__Dialister	0.2388	0.04894	0.2923	0.00949
g__Prevotella	0.366	6.548	3.821	0.01102
g__Lachnospiraceae_UCG-004	0.259	0.1044	0.0274	0.0111
g__Parasutterella	0.5846	0.4665	0.06264	0.01111
g__Ruminococcus_gnavus_group	0.09592	0.3354	0.1312	0.01123
g__Clostridium_sensu_stricto_1	0.3386	0.1266	0.2747	0.0116
g__Oscillibacter	0.04698	0.01109	0.01631	0.0116
g__Morganella	0.0261	0.003915	0.003262	0.01185
g__Desulfovibrio	0.184	0.001305	0.00522	0.01304
g__Vagococcus	0.1312	0.008482	0.0006525	0.01323
g__unclassified_p__Firmicutes	0.007177	0.001957	0.04437	0.01365
g__Ruminococcus_torques_group	0.1553	0.2969	0.261	0.01771
g__Allisonella	0.2388	0.005872	0.04111	0.01778
g__Negativibacillus	0.05742	0.00783	0.001305	0.01908
g__Faecalitalea	0.01762	0.04894	0.0124	0.02034
g__norank_f__Lachnospiraceae	0.201	0.006525	0.003915	0.02106
g__Hungatella	0.01631	0.04567	0.009135	0.02851
g__Anaerostignum	0.004567	0.01044	0.0006525	0.02908

g__Roseburia	0.0137	0.2754	0.02479	0.02982
g__Faecalibacterium	0.171	0.89	0.2068	0.03249
g__Flavonifractor	0.07243	0.03523	0.02806	0.03298
g__Lachnospiraceae_UCG-010	0.0006525	0.01762	0.004567	0.03331
g__Eggerthella	0.2382	0.005872	0.02414	0.03598
g__Weissella	0.01957	22.45	0.1051	0.03782
g__Dorea	0.6173	0.1553	0.07569	0.03884
g__Lachnospira	0.006525	0.07569	0.02414	0.0418
g__Serratia	0.04111	0.009135	0.006525	0.04343
g__Tyzzerella	0.004567	0.01957	0.001957	0.04552
g__Olsenella	0.005872	0.0006525	0.009135	0.04978
g__Parabacteroides	2.27	0.3034	0.2434	0.04998

Table S4. The impact of WMS and MADBS on the abundance of 30 significantly differential bacterial genera in mice.

Species name	CK-mean(%)	MADBS-mean(%)	WMS-mean(%)	P value
g__Allobaculum	9.111	0.4608	6.461	0.0007119
g__Akkermansia	0	0.05516	0.0007355	0.0009173
g__Odoribacter	0.2883	0.02206	0.1636	0.000934
g__Turicibacter	0.2259	0	0.002942	0.002107
g__Parabacteroides	0.2318	0.4862	1.285	0.002703
g__Blautia	0.08179	0.005516	0.009929	0.002707
g__Ileibacterium	3.05	6.845	0.9708	0.003702
g__unclassified_f__Enterobacteriaceae	0.006472	0	0	0.005012
g__Lachnospiraceae_UCG-001	0.03707	0.004413	0.003677	0.005068
g__unclassified_f__Atopobiaceae	0.123	0.7024	0.2074	0.005372
g__norank_f__Ruminococcaceae	6.984	2.117	1.526	0.006119
g__Parasutterella	0.143	0.7421	1.169	0.007435
g__Alistipes	2.983	0.6399	2.547	0.01018
g__norank_f__Muribaculaceae	22.1	49.97	39.89	0.01351
g__Erysipelatoclostridium	0.06766	0.04045	0.4339	0.01661
g__unclassified_f__Rikenellaceae	0.2507	0.0103	0.15	0.01666
g__Roseburia	0.2471	0.0217	0.02979	0.01677
g__Coriobacteriaceae_UCG-002	0	0.0559	0.04339	0.01736
g__Parvibacter	0.0306	0.009929	0.006252	0.01903
g__Rikenella	0.5631	0.2765	0.1427	0.02126
g__Colidextribacter	0.07884	0.008458	0.02942	0.02358
g__Lachnospiraceae_FCS020_group	0.02236	0.002206	0.0007355	0.02377
g__Dubosiella	2.477	5.766	1.471	0.02459
g__Mucispirillum	1.001	0.01986	0.1162	0.03053

g__Gordonibacter	0.6055	0.7002	0.2115	0.03222
g__Eubacterium_nodatum_group	0.006472	0.01066	0.02611	0.03425
g__Dechloromonas	0.002354	0	0	0.03444
g__norank_f__Oscillospiraceae	0.2601	1.289	0.1118	0.04382
g__unclassified_f__Oscillospiraceae	0.7596	0.2765	0.338	0.04517
g__unclassified_o__Coriobacteriales	0.001765	0.01434	0.004045	0.04599
